# A List of Non-Panel Men

**Published:** 1914-06-06

**Authors:** Thomas Johnston

**Affiliations:** 91 Carlisle Road, Hove


*To the Editor of* The Hospital.

### A List of Non-Panel Men.

*To the Editor of* The Hospital.

Sir,—Dr. A. J. Brock admits so much in his letter of the 16th instant that I think there is not much difference between us. Only he does not seem to comprehend that there is a necessity, for the convenience of the public as well as of the profession, that there should be a list of non-panel men available. When a patient of mine removes to another town I am sometimes asked what doctor should be called in. I always prefer to suggest a man who is not on the panel.

I have always found the Hon. Secretaries of the National Medical Union ready to oblige me with the information. This is not so direct as a directory.—I remain, yours faithfully,

Thomas Johnston.
91 Carlisle Road, Hove, May 25, 1914.

